# Roles of ubiquitin in autophagy and cell death

**DOI:** 10.1016/j.semcdb.2018.09.004

**Published:** 2019-09

**Authors:** Carlos Gómez-Díaz, Fumiyo Ikeda

**Affiliations:** Institute of Molecular Biotechnology of Austrian Academy of Sciences (IMBA), Vienna Biocenter, Dr. Bohr-gasse 3, Vienna, Austria

**Keywords:** ATG, autophagy-related, CYLD, cylindromatosis, DUB, deubiquitinase, IAP, inhibitors of apoptosis proteins, LUBAC, linear ubiquitin chain assembly complex, LC3, microtubule-associated protein 1 A/1B-light chain 3, PTM, posttranslational modification, TNF, tumor necrosis factor, TRAIL, TNF-related apoptosis inducing ligand, UBD, ubiquitin-binding domain, UPS, ubiquitin-proteasome system, USP, ubiquitin-specific protease, Autophagy, Cell death, Deubiquitinase, E3 ligase, Inflammation, Ubiquitin

## Abstract

The balance between cell survival and cell death is often lost in human pathologies such as inflammation and cancer. Autophagy plays a critical role in cell survival: essential nutrients are generated by autophagy-dependent degradation and recycling of cellular garbage. On the other hand, cell death is induced by different programs, such as apoptosis, pyroptosis, and necroptosis. Emerging evidence is revealing how cell survival and cell death pathways are coordinated to determine cell fate. For instance, posttranslational modification of proteins with ubiquitin regulates many steps of autophagy and cell death pathways. In this review article, we will discuss how the ubiquitin system influences cell death and autophagy.

## 1 Introduction

The regulation of cell fate by opposing cell death and cell survival pathways is important for cellular and tissue homeostasis [1]. Dysregulation of these signaling pathways can induce pathological conditions such as inflammatory and neuronal diseases [2]. The ubiquitination system plays critical roles in the pathways for cell survival (e.g. autophagy) and cell death (e.g. apoptosis, necroptosis) [[3], [4], [5]]. Furthermore, autophagy and cell death pathways exhibit extensive crosstalk [6,7]. This link is especially well-understood in *Drosophila* [8]. For example, programmed cell death in the *Drosophila* larval midgut tissue is dependent on autophagy, and some autophagy-related (ATG) genes are involved in this process [9]. In mammals, autophagy-dependent cell death is limited and occurs in a context-dependent manner [10,11]. Interestingly, regulators of the ubiquitin system play a role in both cell death and autophagy in mammals, which we will discuss in this review.

**Fig. 1:**
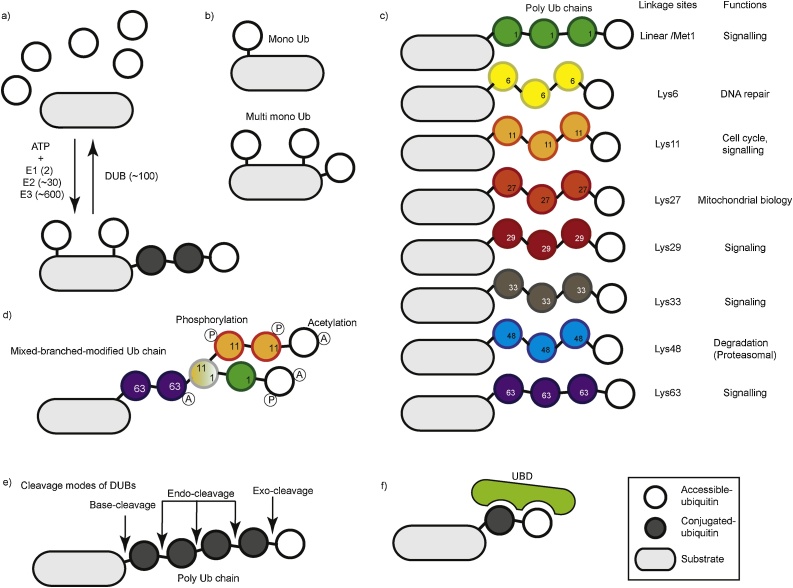
Various types of ubiquitination on a substrate are regulated by distinct enzymes. **a)** Ubiquitination of a substrate via a sequential cascade of ATP-dependent E1 ubiquitin activating enzyme, E2 ubiquitin conjugating enzyme and E3 ligase. A deubiquitinase (DUB) removes ubiquitin (conjugated and accessible distal ubiquitin are indicated as open and filled circles, respectively). **b)** A single ubiquitin attached to a single residue (monoubiquitination, upper) or multiple residues (multi-monoubiquitination, lower). **c)** Substrate ubiquitination with 8 possible linkage types (Met1/linear, Lys6, Lys11, Lys27, Lys29, Lys33, Lys48 and Lys63) of ubiquitin chains. Their major functions are indicated (right). **d)** A hypothetical mixed linkage type ubiquitination (e.g. Met1/linear and Lys63), branched ubiquitination (e.g. Lys11) and with posttranslationally modified ubiquitins (e.g. phosphorylation and acetylation). **e)** Cleavage modes of deubiquitinases (DUBs). Base-cleavage to hydrolyze ubiquitin at the most distal site, endo-cleavage to hydrolyze in between conjugated ubiquitin chains, and exo-cleavage to hydrolyze from the most distal ubiquitin. **f)** A ubiquitin binding domain (UBD)-containing protein recruited to a signaling complex by interacting with a distinct ubiquitin chain.

The 76 amino acid protein ubiquitin is expressed in all cell types and plays a fundamental role in biology [12]. A major function of ubiquitin is to posttranslationally modify substrates via covalent conjugation, typically of Lys residues, although sometimes of Ser, Thr and Cys residues [[13], [14], [15], [16], [17], [18]]. Similar to other types of posttranslational modifications (PTMs), ubiquitination is induced or reversed by enzymes. A unique feature of ubiquitination is that the ubiquitin molecule itself can be modified by PTMs such as phosphorylation, acetylation, sumoylation, neddylation, and ubiquitination [[19], [20], [21]]. The conjugation of a distinct type of ubiquitin chain to a specific substrate residue determines the fate of the substrate, and hence impacts various biological functions. Approximately 1000 ubiquitin system enzymes regulate this complex process (Fig. 1a). Thus, these enzymes play key roles in regulating biological functions via substrate ubiquitination.

In this article, we will describe principles of ubiquitin biology, and subsequently discuss the regulation of autophagy and cell death pathways by ubiquitin system enzymes. We will also discuss how autophagy and cell death may crosstalk directly or indirectly via the ubiquitination system.

## 2 Ubiquitin biology

Ubiquitin is a highly conserved protein that is fundamental to various biological functions. Ubiquitin, which was initially identified as a protein with unknown function in 1975, was later discovered to control energy-dependent intracellular proteolysis [22]. As a result, the most well-understood function of ubiquitin is to target substrate proteins for proteasomal degradation [12]. However, the biological outcomes of ubiquitination depend on how the substrate is modified, and are not restricted to proteasomal degradation of substrates [23]. For instance, ubiquitination can impact the stability, conformation and interactome of substrates.

### 2.1 Ubiquitination and deubiquitination machineries

Substrate ubiquitination is mediated through a cascade of ATP-dependent enzymatic activities, including an E1 activating enzyme, an E2 conjugating enzyme and an E3 ligase (Fig. 1a) [12]. The human genome is known to encode two E1 genes, 30 E2 genes, and over 600 E3 genes [24]. Ubiquitin is a unique PTM that can not only modify a substrate via monoubiquitination (Fig. 1b), but also via polyubiquitination, whereby ubiquitin itself is ubiquitinated at the intrinsic N-terminal Met1 residue and/or at the intrinsic seven Lys residues (Lys6, Lys11, Lys27, Lys29, Lys33, Lys48, Lys63) (Fig. 1c). Polyubiquitination can generate complex ubiquitin chains with distinct lengths, mixed linkage types and branches (Fig. 1d) [21,25]. In some cases, the ubiquitin chain linkage type is determined by the combination of E2 and E3. For example, Met1-linked/linear ubiquitin chains are generated by the “Linear Ubiquitin Chain Assembly Complex” (LUBAC) E3 ligase complex, which is thus far the only known E3 ligase able to generate linear ubiquitin chains [26]. LUBAC determines the linkage, irrespective of the partner E2, and is also predicted to determine substrate selection [[26], [27], [28]]. In contrast, the “Cellular Inhibitor of Apoptosis Protein” 1 and 2 (cIAP1 and cIAP2) ‘Really Interesting New Gene’ (RING)-type E3 ligases [29] generate Lys11-, Lys48- or Lys63-linked ubiquitin chains depending on the E2 [30]. Furthermore, ubiquitin can be phosphorylated, acetylated, neddylated or sumoylated, leading to highly complex ubiquitin modifications (Fig. 1d) [21].

Like other types of PTMs, ubiquitination is a reversible reaction. Approximately 100 deubiquitinating enzymes (DUBs) play a role in this process (Fig. 1a) [31]. DUBs are classified into six families: “Ubiquitin C-terminal Hydrolases” (UCHs), “Ubiquitin-Specific Proteases” (USPs), “Ovarian Tumor Proteases” (OTUs), Josephins and JAB1/MPN/MOV34 metalloenzymes, and “Motif Interacting with Ub-containing Novel DUB familY” (MINDY) [[31], [32], [33]]. Functionally, these DUBs utilize three mechanisms to remove ubiquitin modifications; endo-cleavage occurs between ubiquitin moieties, exo-cleavage occurs from the distal ubiquitin moiety, and base-cleavage removes the entire ubiquitin chain as a block from the substrate (Fig. 1e) [32,34]. Thus, different DUBs, which may display distinct specificities for ubiquitination linkage types, control the removal of ubiquitin chains.

### 2.2 General biological outcomes of ubiquitination

These complex types of ubiquitination have distinct impacts on substrates and, in turn, cellular signaling (Fig. 1c) [23]. It is especially important to consider that the recruitment of specific ubiquitin-binding proteins can regulate signaling complex formation in a spatiotemporal manner. Proteins often utilize a ubiquitin binding domain (UBD) to recognize the ubiquitin code on a substrate (Fig. 1f), and various UBDs have been identified, consistent with the diversity of ubiquitin modifications [19,34,35]. For instance, Rpn1 [36], Rpn10 [36], and Rpn13 [37,38] of the proteasome 19S regulatory particle harbor UBDs specific for Lys48-linked ubiquitin chains, which thus target substrates for proteasome-mediated degradation (Fig. 1c). UBDs are typically within multi-domain and multi-functional proteins, and the conformations of these proteins can be uniquely affected depending on interactions with a single ubiquitin monomer, multiple monomers or ubiquitin chains. Differential binding to ubiquitin moieties has been proposed to regulate the enzymatic activity of UBD-containing E3 ligases like Parkin and HOIL-1-interacting protein (HOIP) [39]. Further, UBD-containing proteins are recruited to signaling or cellular complexes depending on features of the ubiquitin chain. Such cases have been intensively studied in cell death signaling cascades during immune responses [40], as well as in selective autophagy [41,42].

## 3 Ubiquitination in autophagy

**Fig. 2:**
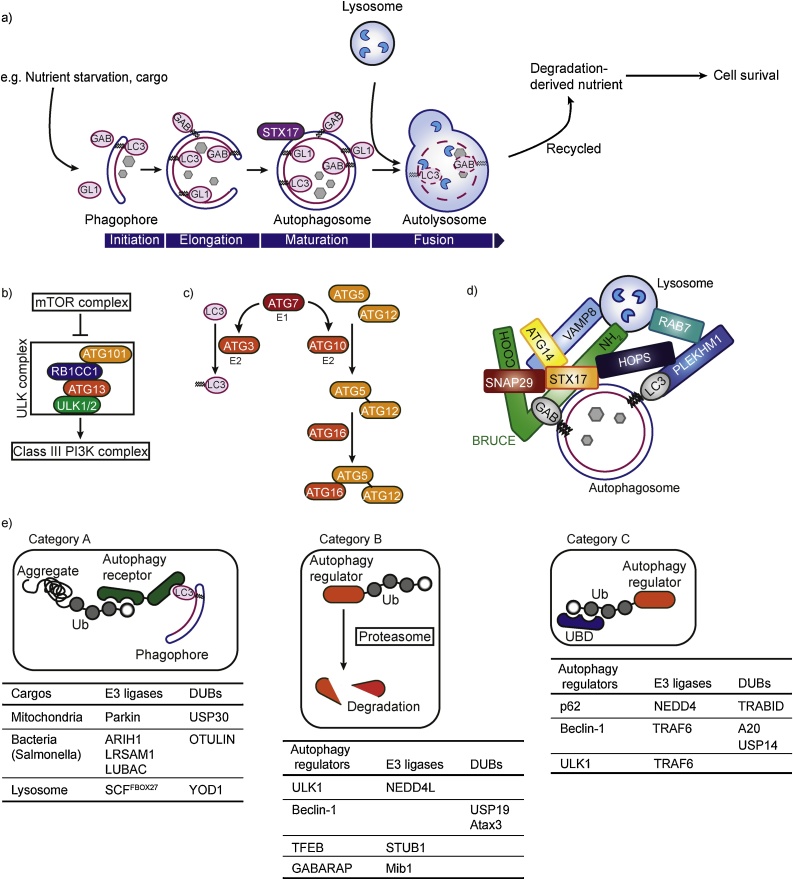
Macroautophagy involves the ubiquitin system. **a)** Macroautophagy induced by nutrient starvation initiates phagophore formation, whereby lipidated LC3, GABARAP (GAB) and GABARAPL-1 (GL1) are components of the elongated double membrane. A mature-closed autophagosome becomes positive for STX17 fuses with a lysosome to form an autolysosome. Cargos are degraded by lysosomal enzymes such as proteases and lipases. Degradation-derived nutrients are recycled back to the cytosol. **b)** The ULK complex, important for autophagy initiation, consists of ATG101, RB1CC1, ATG13 and ULK1/2. The complex is inhibited by the mTOR complex. Inhibition is relieved upon starvation, which activates the downstream class III PI3K complex. **c)** A cascade of LC3 lipidation and ATG5-ATG12-ATG16 complex formation are important for phagophore formation. **d)** Molecules regulating mature autophagosome and lysosome. Lipidated LC3 members including GABARAP (GAB) interacts with Pleckstrin homology domain-containing family M member 1 (PLEKHM1) or BRUCE. Syntaxin 17 (STX17) on mature autophagosome interact with a SNARE protein SNAP29 and Vesicle-associated membrane protein 8 (VAMP8). The HOPS complex and Ras-related protein Rab-7 (RAB7) also play a role. **e)** Three categories of autophagy regulation by the ubiquitin system. Category A: ubiquitinated cargos are recruited to the phagophore via an autophagy receptor with a UBD and a LC3-interacting region (LIR). Category B: autophagy regulators with Lys48-linked ubiquitin chains are targeted for proteasomal degradation. Category C: autophagy regulators with non-degradation-type ubiquitin chains form distinct signaling complexes.

Autophagy is a lysosome-dependent degradation pathway highly conserved from yeast to human, first termed by Christian de Duve in 1963 [43]. There are three types of autophagy, macroautophagy, microautophagy and chaperon-mediated autophagy, which utilize distinct mechanisms to target cargos for degradation by lysosomal enzymes [2]. Critical regulators of macroautophagy include autophagy-related (ATG) proteins and their regulators, initially characterized by genetic approaches in yeast [44]. Macroautophagy (referred to as autophagy hereafter), but not the other two types, is initiated by phagophore formation, where ATGs play a critical role (Fig. 2a–c). Recent findings uncovered roles for the ubiquitin system in the regulation of autophagy [3]. In this section, we will discuss ubiquitin modification types, enzymes, and interacting proteins that regulate autophagy.

### 3.1 Principles of macroautophagy

Autophagy happens at the basal level and can be further increased by various cellular stresses, such as amino acid starvation, bacterial and viral infection, and mitochondrial damage. Once the pathway is triggered, an isolation membrane starts to form a phagophore, which subsequently expands to engulf cargos and form an autophagosome (Fig. 2a) [44]. Fusion of autophagosomes with lysosomes leads to the formation of autolysosomes, where lysosomal hydrolases degrade cargo. Degraded cargos are recycled to provide the cell with nutrients. Thus, autophagy is important not only to eliminate cellular garbage, but also to generate recycled nutrients. Based on these outcomes of autophagy, it is mostly considered to promote cell survival [2].

Autophagy is highly regulated at several levels. Initially, amino acid starvation relieves mTOR-mediated repression of the Unc-51-like autophagy activating kinase 1 (ULK1) complex and the Class III Phosphatidylinositol 3-Kinase Catalytic Subunit Type 3 (PI3KC3)/VPS34 complex, which are critical for phagophore nucleation (Fig. 2b) [10]. Alternatively, cargos can induce autophagosome formation in a type of autophagy called “selective autophagy” [[45], [46], [47], [48], [49]]. Subsequently, phagophore elongation occurs by the ATG12-ATG5-ATG16L1 complex, and LC3-lipidation (Fig. 2c) [44]. Ultimately, an autophagosome is formed, enclosing cargos inside. Mature autophagosomes recruit Syntaxin 17 and SyNaptosome Associated Protein 29 (SNAP29), and fuse with Vesicle Associated Membrane Protein 8 (VAMP8) on lysosomes (Fig. 2d) [50]. The HOPS complex, PLEKHM1, Rab7, as well as ATG14, also play important roles in autophagosome-lysosome fusion (Fig. 2d) [51]. How the dynamic process of autophagy is precisely regulated at the molecular level remains of interest.

### 3.2 The ubiquitin system in autophagy

Ubiquitin is involved in many aspects of autophagy [3,52]. For example, aggregated proteins, bacteria (exclusively *Salmonella*) and damaged mitochondria tagged with ubiquitin chains can be brought to the autophagosomal membrane via an autophagy receptor [42], and thus represent cargos targeted for autophagy-dependent degradation (Fig. 2e, Category A). In addition, autophagy regulators such as ULK1, VPS34 and Beclin-1 can be ubiquitinated and degraded by the ubiquitin proteasome system (UPS), which in turn directly affects autophagic flux (Fig. 2e, Category B) [[53], [54], [55]]. Ubiquitin tags on autophagy regulators, such as LC3 family members [56], may also regulate autophagy by influencing protein folding and interactomes (Fig. 2e, Category C). For instance, a newly developed polyubiquitin-mediated fluorescence complementation (PolyUb-FC) assay revealed that the interaction between autophagy receptor p62 and Lys33-linked ubiquitin chains at the autophagosome is important for autophagosome formation [57].

Mice with tissue-specific autophagy-defects accumulate protein aggregates tagged with Lys11-, Lys48- and Lys63-linked chains, further suggesting a link between ubiquitination and autophagy [58]. This is contrary to the previously-speculated concept that protein aggregates tagged with Lys48-linked chains, that are not degraded by proteasome, are simply cleaned up by the autophagy-lysosomal pathway. These data rather suggest that autophagy cargos are selected based on different types of ubiquitination. In line with this, not only Lys48-linked chains but also Met1- and Lys63-linked ubiquitin chains were found to be conjugated on cytosolic *Salmonella* for their clearance by autophagy (xenophagy) [59].

Historically, the lysosome was believed to be the only degradation machinery in cells. Subsequently, the proteasome was identified as a degradation machine that depends on ubiquitin [22]. Conceptionally, it is intriguing that ubiquitin regulates not only the UPS but also autophagy-dependent lysosomal degradation of cargos [60].

### 3.3 Ubiquitin-related enzymes, E3 ubiquitin ligases and DUBs in autophagy

The most well-understood autophagy pathway involving ubiquitin is selective autophagy. This pathway can target selected cargos conjugated with ubiquitin chains for autophagy-dependent degradation via Category A (Fig. 2e, Category A), including protein aggregates (aggrephagy), damaged mitochondria (mitophagy), lysosome (lysophagy) and bacteria (xenophagy).

Mitophagy can be regulated by the E3 ligase Parkin, which is activated by phosphorylation via PTEN-INduced putative Kinase 1 (PINK1) [41]. PINK1 also phosphorylates ubiquitin at the mitochondria. Activated Parkin generates ubiquitin chains linked via Lys6, Lys11, Lys48 and Lys63 on the mitochondrial outer membrane (MOM) of damaged mitochondria. The DUB USP30 reverses this polyubiquitination and thus can inhibit mitophagy [61,62]. It remains unclear why and when USP30 would inhibit the removal of damaged mitochondria via mitophagy. Alternatively, USP30 might function to maintain the basal levels of ubiquitination at MOM.

Xenophagy clears ubiquitin-coated cytosolic bacteria (exclusively *Salmonella*) via autophagy [63]. Until recently, it was largely unclear which enzymes are responsible for the ubiquitination of *Salmonella* during xenophagy; however, an RNAi screen revealed that the E3 ligase ARIH1/HHARI is required to ubiquitinate cytosolic *Salmonella* [59]. Of note, ATG7 deficiency further inhibits clearance of *Salmonella* in ARIH1-deficient cells, suggesting that xenophagy proceeds through ARIH1-independent/ATG7-dependent and ARIH1-dependent mechanisms [59]. The E3 ligase LRSAM1, previously identified as a xenophagy regulator, colocalizes with ARIH1 on cytosolic *Salmonella*, implying that these two E3 ligases might function cooperatively [64]. The E3 ligase complex LUBAC was also shown to be important in xenophagy; LUBAC is required to form linear ubiquitin chains on the surface of *Salmonella* in the cytosol, thereby activating NF-κB signaling and restricting proliferation of *Salmonella* [65]. The linear ubiquitin chain-specific DUB OTULIN negatively regulates this process [66].

Lysophagy also depends on the ubiquitin system [67]. Here, the ubiquitin E3 ligase SCF^FBXO27^ ubiquitinates Lysosome-associated membrane protein 2 (LAMP2) when the lysosome is damaged and ruptured, leading to autophagy-dependent degradation. Removal of Lys48-linked ubiquitin chains by the DUB YOD1, a component of the endo-lysosomal damage response pathway (ELDR) complex, is also critical for lysophagy [68]. Without the ELDR complex, Lys48- and Lys63-linked ubiquitin chains on damaged lysosomes accumulate in cells, suggesting that ELDR directly or indirectly regulates this process by cleaving those chains.

Ubiquitination of autophagy regulators often affects their stability (Fig. 2e, Category B). The E3 ligase STIP1 Homology And U-Box Containing Protein 1 (STUB1)/CHIP in the mTOR-autophagy pathway ubiquitinates the transcription factor Transcription Factor EB (TFEB), a major transcriptional regulator of lysosome biogenesis genes, leading to UPS-dependent degradation [69]. In addition, the E3 ligases NEDD4L and Mib1 induce UPS-dependent degradation of ULK1 and GABARAP, respectively [54,55]. The DUB Atax3 hydrolyzes Lys48-linked ubiquitin chains on Beclin-1, thus promoting the stability of this essential autophagy initiator [70]. Beclin-1 is also regulated by USP19, which removes conjugated Lys11-linked ubiquitin chains and thereby stabilizes Beclin-1 to positively regulate autophagy [71].

Ubiquitin can also be utilized as a UPS-independent tag to regulate autophagy (Fig. 2e, Category C). For example, the HECT-type E3 ligase NEDD4 mediates ubiquitination of p62 with Lys63-linked chains, which are not involved in UPS but rather in inclusion body autophagy [72]. In another case, the DUB Zinc Finger RANBP2-Type Containing 1 (ZRANB1)/ TRABID cleaves Lys33-linked ubiquitin chains conjugated on the p62 complex to regulate autophagy [57]. Additionally, the DUB USP14 is activated via AKT-mediated phosphorylation and subsequently cleaves Lys63-linked ubiquitin chains conjugated on Beclin-1 [73]. Lys63-linked ubiquitin chain conjugation on Beclin-1 is also induced by TRAF6 in the Toll-Like 4 (TLR4)-induced autophagy pathway, which is reversed by the DUB A20 [74]. Recent screens for novel autophagy regulators have uncovered enzymes in the ubiquitin system, such as the E2/E3 hybrid enzyme BRUCE [75]. BRUCE regulates the fusion of mature autophagosomes with lysosomes during amino acid starvation-dependent autophagy, potentially independently of its enzymatic activity. The RING-type ubiquitin E3 ligase XIAP, which belongs to the same inhibitor of apoptosis (IAP) family as BRUCE, was also shown to be important for autophagosome-lysosome fusion [76]. Thus, many ubiquitin-regulating enzymes control autophagy. This interplay between autophagy and the ubiquitin system requires further investigation.

## 4 Ubiquitination in cell death

Cell death pathways are tightly regulated in a spatiotemporal manner to control homeostasis *in vivo*, and are controlled by extrinsic and intrinsic stimuli. The ubiquitin system plays important roles at various steps in the diverse mechanisms that regulate cell death. In this section, we describe how the ubiquitin system regulates cell death by focusing on ubiquitinating enzymes, E2s and E3s, as well as DUBs.

### 4.1 The ubiquitin system in the extrinsic cell death pathways

Different types of ubiquitin conjugates function in the extrinsic cell death signaling cascades induced by Tumor necrosis factor (TNF) family members, TNF, TNF-related apoptosis-inducing ligand (TRAIL), TNF-related weak inducer of apoptosis (TWEAK) and Fas ligand (FasL). Among them, TNF is unique as it regulates both survival (via TNFR complex I) and death pathways (via TNFR complex II and necrosome) whereas the other ligands mainly trigger cell death. Here, we discuss the major role of ubiquitin in the TNF-pathway.

**Fig. 3:**
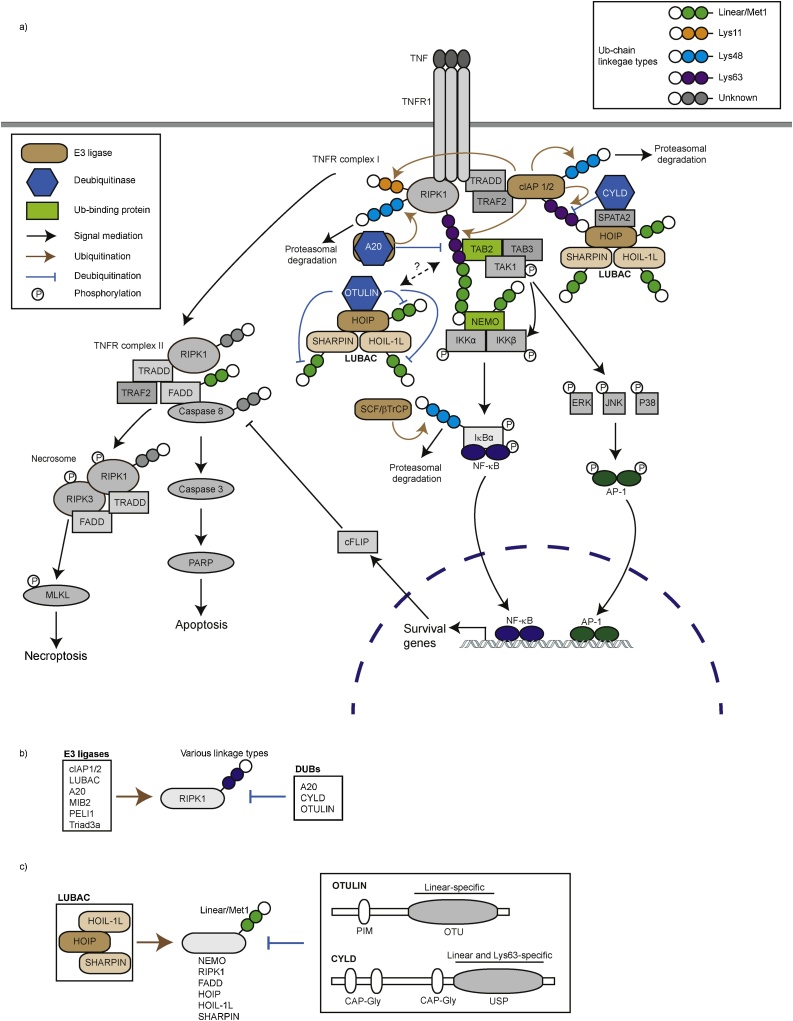
TNF-dependent signaling cascade involving the ubiquitin system. **a)** Binding of TNF to TNF-Receptor 1 (TNFR1) triggers TNFR complex I formation. Diverse E3 ubiquitin ligases (in brown) generate distinct ubiquitin linkage chains that promote the recruitment of specific signaling molecules. For example, RIPK1 is ubiquitinated by cIAP1/2 with Lys11-, and Lys63-linked ubiquitin chains. LUBAC is recruited to TNFR dependently on cIAP activity, where it adds linear ubiquitin chains on RIPK1. The E3 ligase-DUB hybrid A20 ubiquitinates RIPK1 and cleaves Lys63-linked chains on RIPK1. DUBs such as CYLD and OTULIN hydrolyze ubiquitin chains on cIAPs and LUBAC. IKK phosphorylates an inhibitory factor IκB-α which leads to its ubiquitination for proteasomal degradation. Released transcription factors, NF-κB, nuclear translocate and induce transcription of pro-survival genes. Destabilization of complex I leads to formation of complex II, where RIPK1, Caspase 8 and FADD are ubiquitinated. TNFR complex II induces apoptosis and necrosome induces necroptosis. **b)** E3 ligases for RIPK1 (A20, Triad3, cIAP1/2, MIB2, and PELI) regulating cell death. **c)** LUBAC consists of HOIP with a catalytic region, HOIL-1 L and SHARPIN. LUBAC generates linear ubiquitin chains on substrates (NEMO, FADD, HOIP, HOIL-1 L and SHARPIN). The DUBs OTULIN (linear linkage specific) and CYLD (linear and Lys63-linkage specific) cleave linear ubiquitin chains.

Both degradation (Lys48-linked) and non-degradation (Met1-, Lys11- and Lys63-linked) ubiquitin chain modifications regulate the TNF signaling cascade (Fig. 3a). TNF stimulation leads to recruitment of TRADD, TRAF2 and RIPK1 to TNF receptor 1 (TNFR1), forming (TNFR) complex I (Fig. 3a). Subsequently, the E3 ligases IAPs and LUBAC join TNFR complex I and ubiquitinate RIPK1 with linear/Met1-, Lys11- and Lys63-linked ubiquitin chains. These modifications recruit the kinase complexes TAK1 and IKK via UBDs within TAB2 and NEMO, respectively (Fig. 3a) [77,78]. TNFR complex I formation not only activates the pro-survival NF-κB pathway, inducing anti-apoptotic genes such as cFLIP, but also inhibits formation of the cell death mediator complex, TNFR complex II, which consists of TRAF2, FADD, TRADD, RIPK1 and Caspase 8. In the TNFR complex II, IAPs ubiquitinate RIPK1 and Caspase 8 [79].

RIPK1 ubiquitination plays a critical role at the cell death check point (Fig. 3a). Many E3 ligases have been shown to ubiquitinate RIPK1 (Fig. 3b); MIB2 ubiquitinates RIPK1 to inhibit cell death via the TNF pathway [80], Triad3a ubiquitinates and degrades the LPS-induced necrosome components RIPK1 and Caspase 8 [81], and PELI1 ubiquitinates RIPK1 with Lys63-linked chains to inhibit necroptosis [82]. RIPK1 is an excellent example of a substrate for ubiquitination as well as phosphorylation, and the interplay between these two types of PTMs is a critical determinant of cell fate (thoroughly discussed in previous reviews) [[83], [84], [85], [86]].

The E3 ligase complex LUBAC plays a key role in cell death signaling. LUBAC consists of the catalytic core protein HOIP and two subunits SHARPIN and HOIL-1 L (Fig. 3c). Mice harboring a spontaneous gene mutation in *Sharpin,* called ‘chronic proliferative dermatitis mice’ (CPDM), exhibit massive keratinocyte apoptosis and develop systemic inflammation including skin inflammation [[87], [88], [89]]. The apoptosis and inflammation phenotypes in *Sharpin^cpdm/cpdm^* skin were attenuated in crosses with *Tnf*^−/−^, *Tnfr1*^−/−^, epidermis-specific *Fadd*^−/−^; *Ripk3*^−/−^, epidermis-specific *Tradd*^−/−^, or RIPK1-inactive mutant knockin mice, indicating that TNF-dependent apoptosis is the major driver of the phenotype [88,90,91]. Other LUBAC components, HOIP and HOIL-1 L, also regulate cell death [92]. *Hoip*^−/−^ and *Hoil-1l*^−/−^ mice are embryonically lethal at embryonic stage E10.5 with massive endothelial cell death mediated by TNFR1; catalytically inactive HOIP knockin mice are also embryonically lethal [[93], [94], [95], [96]]. FADD is ubiquitinated by LUBAC and this modification is decreased under pro-apoptotic conditions [97]. RIPK1 displays linear ubiquitination [88], and given that LUBAC is the only known E3 ligase for linear ubiquitination, RIPK1 has been proposed as a substrate of LUBAC. Thus, LUBAC may regulate cell death by affecting TNFR complex II formation. The inflammatory phenotypes of *Sharpin^cpdm/cpdm^* mice, including cell death, are rescued by genetic abrogation of Caspase 1, a regulator of pyroptosis [98]. Because SHARPIN regulates the NLRP3 inflammasome [99], which is known to mediate pyroptosis, these studies suggest a link between LUBAC and pyroptotic cell death. HOIP regulates TLR3- and TRAIL-induced death by restricting the formation of the death-inducing signaling complex (DISC) [100,101]. Collectively, these findings establish the E3 ligase complex LUBAC as a key negative regulator of cell death.

Two DUBs, CYLD and OTULIN, form distinct complexes with LUBAC via HOIP (Fig. 3a) [[102], [103], [104], [105]]. CYLD hydrolyzes Lys63-linked and linear ubiquitin chains [106], whereas OTULIN is specific for linear ubiquitin chains (Fig. 3a, c) [107,108]. CYLD and OTULIN antagonize linear ubiquitin chains induced by LUBAC, thereby inhibiting LUBAC-dependent downstream signaling pathways (Fig. 3b and c). CYLD is known to be involved in TNFR1-mediated apoptosis and necroptosis [109,110]. Although biochemical properties are shared between CYLD and OTULIN regarding linear ubiquitin chain hydrolysis, their physiological roles are very distinct. CYLD deficient mice show deficient T cell development [111] and osteoporosis [112]. On the other hand, OTULIN-deficient Gumby mice as well as OTULIN catalytic inactive knockin mice are embryonically lethal at the stage of E10.5- E14, where massive cell death is observed in yolk sac and placenta [107,113]. OTULIN was shown to counteract LUBAC auto-ubiquitination to suppress cell death [113], further indicating important role of linear ubiquitination in cell death regulated by OTULIN and LUBAC.

A20, a hybrid deubiquitinase and E3 ligase, negatively regulates downstream TNF signaling by hydrolyzing Lys63-linked polyubiquitin chains conjugated on RIPK1 and by conjugating Lys48-linked ubiquitin chains on RIPK1, via its OTU and ZF4 domains, respectively (Fig. 3a) [114]. A20 also inhibits the LUBAC-dependent NF-κB pathway by interacting with linear ubiquitin chains through its ZF7 domain [112,115]. A20 knockout mice (*Tnfaip3*^−/−^) display systemic inflammation and hyper sensitivity to TNF, which is in line with the observations in cells [116].

It will be critical to elucidate how the activities of ubiquitin E3 ligases and DUBs are coordinated to control ubiquitination of cell death regulators *in vivo*.

### 4.2 The ubiquitin system in the intrinsic cell death pathways

**Fig. 4:**
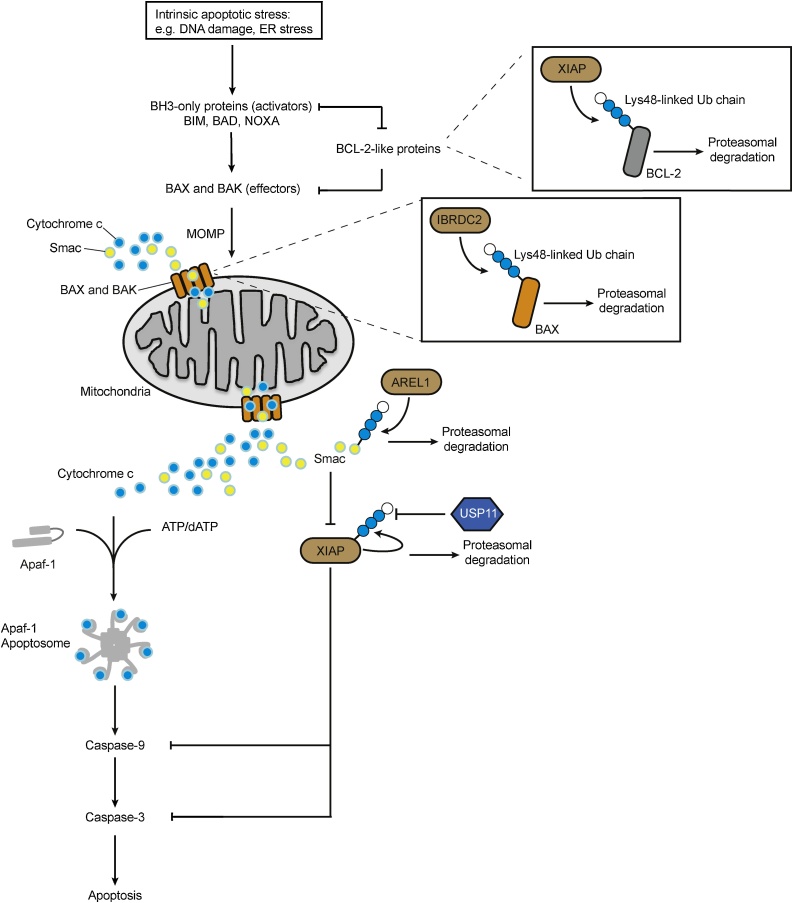
Ubiquitin regulates the intrinsic apoptotic pathway. Upon intrinsic apoptotic stress, mitochondrial membrane integrity is compromised regulated via the BH3-only proteins, BCL-2-like proteins and BAX, BAK effectors. BCL-2 can be ubiquitinated by XIAP for proteasomal degradation. Mitochondrial Outer Membrane Permeability (MOMP) leads to the release of pro-apoptotic molecules, such as Cytochrome c, which promote the formation of the death-inducing Apoptosome complex. The E3 ligase IBRDC2 antagonizes cell death by conjugating Lys48-linked ubiquitin chains onto BAX, a critical molecule for MOMP. Apoptosome activates Caspases leading to apoptosis. The pro-apoptotic molecules Smac are ubiquitinated by XIAP or AREL1 with Lys48-linked chains, targeting it for UPS-dependent degradation. The anti-apoptotic XIAP E3 ligase self-ubiquitinates with Lys48-linked ubiquitin chain which is antagonized by the DUB USP11, regulating the protein stability of XIAP.

Various cellular stresses, such as DNA damage, induce cell death through the intrinsic pathway (Fig. 4). Here, the mitochondria act as a hub to integrate survival and death signals. Engagement of the intrinsic pathway occurs when internal damage is irreparable, leading to the permeabilization of the mitochondrial membrane and the release of several pro-apoptotic molecules from the mitochondrial intermembrane space, including cytochrome c and the IAP antagonist SMAC/Diablo (Fig. 4). Cytochrome c promotes formation of the apoptosome, a large complex consisting of Apaf-1, Cytochrome c and dATP. Subsequently, the apoptosome activates Caspase 9, leading to the activation of effector caspases, Caspase 3 and Caspase 7. E3 ubiquitin ligases control the stability of regulators. For example, Apoptosis Resistant E3 Ubiquitin Protein Ligase 1 (AREL1) ubiquitinates SMAC/Diablo for UPS-dependent degradation [117]. The E3 ligases XIAP and IBRDC2 ubiquitinate the anti-apoptosis BCL-2 members (such as Bcl-2, Bcl-xL and Mcl-1), which are the sensors of intracellular stress [118,119]. It was also shown that the transmembrane E3 ligase RNF183 mediates the ER-induced apoptosis pathway by ubiquitinating Bcl-xL [120]. Mcl-1 is ubiquitinated by various E3 ligases, including the HECT-type E3 ligase Mule (Mcl-1 ubiquitin ligase E3), TRIM17, MULE, SCFβ−TrCP, SCFFBW7, and APC/CCdc20, all of which trigger proteasomal degradation leading to cell death [121].

## 5 Ubiquitination in autophagy and cell death

There are at least two possible ways that autophagy and cell death may cross talk via the ubiquitin system. First, the autophagy pathway can target various cell death-regulating ubiquitin enzymes/ligases for lysosomal degradation. For example, in *Drosophila*, the anti-apoptotic E2 enzyme dBruce is degraded by autophagy during late oogenesis [122]. Thus, it is speculated that autophagy influences apoptosis in this context by regulating the levels of dBruce. Second, ubiquitin system-related factors, which regulate cell death, can also control autophagy. For instance, the E3 ligase TRAF6 regulates the extrinsic pathway in an anti-apoptotic manner, but also ubiquitinates the autophagy regulator kinase ULK1 by forming a complex with AMBRA1 [123]. Additionally, the linear ubiquitin chain-binding protein Optineurin is important for both anti-apoptosis and for selective autophagy as an autophagy-receptor [124]. In such cases, regulation of TRAF6 or Optineurin determines whether it promotes cell death or autophagy.

From the perspective of cellular organelles, mitochondria are key in coordinating the pathways of cell death and autophagy. The Beclin-1/Bcl-2 interacting protein AMBRA-1 [125] regulates intrinsic apoptosis by mediating the interaction with Cullin E3 ligases [126], and autophagy by mediating the interaction with the Parkin E3 ligase [127]. It will be important to understand how these distinct AMBRA-1-E3 ligase complexes are formed and activated.

Finally, there is a type of cell death called autophagic cell death, which mechanistically depends on the autophagic machinery [6,11]. In general, autophagy is utilized for cell survival, however autophagy can induce regulated-cell death in some specific tissues in different species. In *Drosophila*, it is well understood from genetic studies that autophagic cell death is critical to remove tissues during development [128]. Further studies are required to understand whether and how the ubiquitin system may be involved in regulation of autophagic cell death.

## 6 Conclusion

Many enzymes within the ubiquitin system regulate both autophagy and cell death, and might coordinate these cell fate decisions by controlling distinct substrates or ubiquitin modifications (summarized in Table 1). A very recent study showed that the E3 ligase MIB2 was identified in a screen for RIPK1-interacting proteins; this screen also uncovered the well-known autophagy receptors, p62 and NBR1 [80]. RIPK1 is a key ubiquitination substrate in the immune signaling cascade, and its ubiquitination status may help control the balance between cell death and autophagy.

**Table 1:** Ubiquitin system-related proteins with dual roles in autophagy and cell death.

Proteins	Functions in autophagy	Functions in cell death	Ubiquitin-related roles
XIAP	Promotes autophagosome-lysosome fusion.	Inhibits apoptosis by Caspase 3/7. Ubiquitinates anti-apoptosis BCL-2 members.	The IAP E3 ligase family member (E3)
BRUCE	Promotes autophagosome-lysosome fusion. In Drosophila, degraded by autophagy.	Inhibits apoptosis.	The IAP E3 ligase family member (E2)
LUBAC	Regulates Salmonella clearance by selective autophagy.	Inhibits the extrinsic cell death pathways (TNF, TRAIL)	Linear ubiquitin chain-specific E3 ligase complex
OTULIN	Regulates Salmonella clearance by selective autophagy by antagonizing LUBAC.	Regulates cell death by antagonizing LUBAC.s	Linear ubiquitin chain-specific DUB
TRAF6	Ubiquitinates ULK1.	Inhibits extrinsic apoptosis.	E3 ligase
OPTINEURIN	Autophagy receptor.	Inhibits extrinsic apoptosis.	Linear ubiquitin chain-specific-binding protein
BCL-2	Inhibits autophagy by interacting with Beclin-1.	Inhibits intrinsic apoptosis.	Substrate
BAX	Inhibits autophagy by promoting cleavage of Beclin-1.	Induces intrinsic apoptosis by disrupting the mitochondrial membrane.	Substrate

Both autophagy and cell death influence human pathologies such as inflammation and cancer. Understanding how the ubiquitin system regulates cell death and autophagy might reveal novel therapeutic targets and strategies to treat human diseases.

## Funding

Research in Ikeda Lab is supported by the FWF standalone grant, Austrian science fund [P 25508]; the ERC consolidator grant [LUbi, 614711]; the COST, European Cooperation in Science and Technology [PROTEOSTASIS BM1307]; and the Austrian Academy of Sciences.
